# Application of type 2 inflammation-related biomarkers in risk prediction models for acute exacerbations in adult asthma: a systematic review

**DOI:** 10.3389/fmed.2026.1900214

**Published:** 2026-09-18

**Authors:** Minghao Li, Zihan Yan, Minghang Wang

**Affiliations:** 1 Lung Disease Diagnosis and Treatment Center, National Medical Center, The First Affiliated Hospital of Henan University of Chinese Medicine, Zhengzhou, China; 2 Collaborative Innovation Center for Chinese Medicine Prevention and Treatment of Respiratory Diseases Co-constructed by Province and Ministry, Zhengzhou, China

**Keywords:** acute exacerbation, asthma, blood eosinophils, FeNO, IgE, prediction model, systematic review, type 2 inflammation

## Abstract

**Objective:**

To systematically evaluate how type 2 (T2) inflammation-related biomarkers are incorporated into multivariable models that predict future acute exacerbations in adults with asthma, and to appraise model performance, validation, calibration, risk of bias, and clinical applicability.

**Methods:**

PubMed, Embase, Web of Science, the Cochrane Library, and Scopus were searched from inception to May 2026, followed by a supplementary PubMed update through 31 July 2026. Eligible reports contained at least one adult cohort used to develop, validate, or update a longitudinal multivariable model for a future exacerbation outcome, incorporated at least one T2-related biomarker, and reported model performance or validation. Mixed-age cohorts were not counted as independent adult evidence; only performance evaluated in an adult cohort contributed to the main synthesis. Association-only studies, current-attack classification models, post-exacerbation disposition models, and post-index relapse models outside the prespecified baseline-risk setting were excluded. Data extraction followed CHARMS, and risk of bias and applicability were assessed using PROBAST.

**Results:**

Eight reports published from 2021 to 2026 were included. Eligible adult cohorts ranged from 540 to 1,331,934 participants; one report also included a mixed-age randomized-trial comparator (*n* = 345). Modeling approaches included logistic and elastic-net regression, gradient-boosting and deep-learning algorithms, Bayesian networks, and a mixed-effects count model. Eosinophil-related variables appeared in all eight reports but were not retained in every final model; FeNO was included in two multinational severe-asthma registry models, and serum IgE appeared as a network node in one cross-source analysis and was retained in an independently replicated metabolomics model. The metabolomics model reported AUCs of 0.901 and 0.893 in discovery and replication cohorts, whereas an RCT-derived Bayesian network achieved an AUC of 0.68 when validated in the adult ISAR cohort. Six reports were rated at high overall risk of bias, mainly because of analysis-domain limitations.

**Conclusion:**

T2-related biomarkers may add complementary information to adult asthma exacerbation-risk models, but the available evidence does not yet establish BEC, FeNO, or IgE as independently sufficient predictors or support routine implementation of an existing model. Future studies should quantify incremental biomarker value beyond established clinical predictors and prioritize adequate sample size, external validation, calibration, clinical utility, transparent handling of missing biomarker data, and reproducible model presentation.

**Systematic Review Registration:**

https://www.crd.york.ac.uk/prospero/, PROSPERO CRD420261415469.

## 1 Introduction

Asthma is a heterogeneous chronic airway disease characterized by variable respiratory symptoms and airflow limitation (1). Acute exacerbations are clinically important events that may require systemic corticosteroids, emergency care, hospitalization, or intensive care, and contribute substantially to morbidity and health-care use (2, 3). Accurate risk estimation could support targeted follow-up, adherence interventions, optimization of anti-inflammatory therapy, and timely specialist referral.

T2 inflammation is a prominent inflammatory endotype in asthma involving IL−4, IL-5, and IL-13 signaling, eosinophilic inflammation, and IgE-associated responses (4, 5). Blood eosinophil count (BEC) and fractional exhaled nitric oxide (FeNO) are among the most accessible clinical indicators of this biology, whereas total IgE and periostin have less consistent roles in general exacerbation-risk assessment (6). Biomarker interpretation is context dependent because values may vary with corticosteroid exposure, infection, allergic comorbidity, smoking, treatment adherence, and measurement timing. A single measurement or fixed threshold may therefore be insufficient for individualized risk estimation across heterogeneous asthma populations.

A prognostic prediction model differs from a biomarker-association study. It combines multiple predictors to estimate an individual's future probability or rate of an outcome and should report discrimination, calibration, validation, or clinical utility (7). An adjusted odds ratio or hazard ratio may show prognostic association without yielding a transportable individual-risk tool. Previous systematic reviews synthesized asthma-attack prediction models in primary care and broader adult populations (8, 9), but did not specifically evaluate the measurement, modeling, reporting, and incremental value of T2-related biomarkers. This review therefore focuses on adult multivariable exacerbation-risk models that incorporate at least one T2-related biomarker and examines apparent performance alongside methodological credibility.

## 2 Materials and methods

### 2.1 Reporting guideline and registration

This systematic review was conducted in accordance with PRISMA 2020 (10), and the protocol was registered in PROSPERO (CRD420261415469). Before completion of the updated screening, the eligibility framework was operationally clarified to distinguish individualized prognostic models from association-only studies and to define the prespecified baseline-risk setting; this clarification was documented as a protocol amendment.

### 2.2 Eligibility criteria

Reports were eligible when they: (1) contained an adult cohort aged 18 years or older with established asthma, or extractable adult-only development or validation results; (2) used a longitudinal design in which predictors were measured before the target outcome; (3) developed, validated, or updated a multivariable model for a future asthma exacerbation, severe exacerbation, asthma-related emergency visit or hospitalization, or systemic-corticosteroid-treated attack, with prediction initiated from a baseline distinct from the target event and not during the same unresolved exacerbation; (4) incorporated at least one T2-related biomarker, including BEC/AEC, FeNO, IgE, periostin, or an explicitly defined T2-biomarker construct, as a predictor, network node, or prespecified stratification variable; and (5) reported discrimination, calibration, classification performance, internal or external validation, or clinical net benefit. Prototype and *post hoc* tools were eligible only when adult participant-level data produced individualized multivariable risk estimates with performance evaluation. Reports with an eligible adult cohort could include a mixed-age auxiliary cohort. Only performance evaluated in an adult cohort contributed to the main synthesis; results evaluated solely in a mixed-age cohort were contextual and were not counted as independent adult evidence.

Reports were excluded when they reported biomarker-outcome associations without an individualized multivariable prediction output; evaluated only a single biomarker threshold; detected or classified a current exacerbation; predicted severity, immediate disposition, hospitalization, treatment failure, or short-term return for the same unresolved exacerbation; used an index exacerbation as the prediction origin and targeted a subsequent relapse composite outside the prespecified baseline-risk setting; predicted incident asthma; lacked an eligible T2 biomarker; provided no extractable adult development or validation evidence; or were cross-sectional studies, conference abstracts, reviews, commentaries, case reports, or basic research. Aggregate subgroup risk scales and *post hoc* association analyses without an individualized validated tool were also excluded. Table 1 summarizes this operational boundary.

**Table 1:** Operational boundary between eligible prognostic models and excluded association or classification studies.

Domain	Eligible study	Excluded study
Temporal sequence	Predictors measured before a distinct future exacerbation	Concurrent assessment or current-attack classification
Model output	Individual risk, score, equation, or algorithm	Adjusted OR/HR/RR only
Biomarker role	T2 biomarker in the model or prespecified stratum	Isolated exposure or threshold only
Performance	Discrimination, calibration, validation, or net benefit	No performance or validation metric
Clinical target	Future exacerbation from a baseline outside the same acute episode	Incident asthma, same-attack disposition, or post-index relapse composite
Prototype/*post hoc* tool	Adult participant-level individualized model with performance evaluation	Aggregate strata, association-only analysis, or no validation
Population	Adult-only cohort or extractable adult development/validation evidence	No extractable adult evidence; mixed-age data only

HR, hazard ratio; OR, odds ratio; RR, risk ratio; T2, type 2. Mixed-age cohorts were not counted as independent adult evidence; only performance evaluated in an adult cohort contributed to the main synthesis.

### 2.3 Information sources and search strategy

PubMed, Embase, Web of Science, the Cochrane Library, and Scopus were searched from database inception through 31 July 2026. Search concepts covered asthma, future exacerbation-related outcomes, T2-related biomarkers, and prediction-model terminology. Controlled vocabulary and free-text terms were adapted for each database, and reference lists of included studies and relevant systematic reviews were screened. Search reporting followed PRISMA-S principles (11). The updated PubMed strategy is presented in Table 2.

**Table 2:** Search strategy for the pubMed database.

Search number	Search strategy
#1	“Asthma”[Mesh] OR asthma[Title/Abstract] OR bronchial asthma[Title/Abstract] OR asthmatic[Title/Abstract]
#2	"Disease Progression"[Mesh] OR exacerbat*[Title/Abstract] OR “acute attack"[Title/Abstract] OR “acute attacks"[Title/Abstract] OR “asthma attack"[Title/Abstract] OR “asthma attacks"[Title/Abstract] OR worsen*[Title/Abstract] OR hospitali*[Title/Abstract] OR “emergency department"[Title/Abstract] OR “emergency departments"[Title/Abstract] OR “emergency visit"[Title/Abstract] OR “emergency visits"[Title/Abstract] OR “oral corticosteroid"[Title/Abstract] OR “oral corticosteroids"[Title/Abstract] OR “systemic corticosteroid"[Title/Abstract] OR “systemic corticosteroids"[Title/Abstract] OR glucocorticoid*[Title/Abstract]
#3	"Models, Statistical"[Mesh] OR predict*[Title/Abstract] OR prognos*[Title/Abstract] OR model*[Title/Abstract] OR “risk prediction"[Title/Abstract] OR “risk assessment"[Title/Abstract] OR “prediction model"[Title/Abstract] OR “prediction models"[Title/Abstract] OR “prognostic model"[Title/Abstract] OR “prognostic models"[Title/Abstract] OR “risk model"[Title/Abstract] OR “risk models"[Title/Abstract] OR “risk score"[Title/Abstract] OR “risk scores"[Title/Abstract] OR “risk index"[Title/Abstract] OR “risk indices"[Title/Abstract] OR “risk calculator"[Title/Abstract] OR “risk calculators"[Title/Abstract] OR nomogram*[Title/Abstract] OR “machine learning"[Title/Abstract] OR “artificial intelligence"[Title/Abstract] OR “deep learning"[Title/Abstract] OR “logistic regression"[Title/Abstract] OR “Cox regression"[Title/Abstract] OR “proportional hazards"[Title/Abstract] OR Lasso[Title/Abstract] OR “random forest"[Title/Abstract] OR “gradient boosting"[Title/Abstract] OR “Bayesian network"[Title/Abstract] OR “Bayesian networks"[Title/Abstract] OR validation[Title/Abstract] OR calibration[Title/Abstract] OR discrimination[Title/Abstract] OR AUC[Title/Abstract] OR “decision curve"[Title/Abstract]
#4	"Eosinophils"[Mesh] OR “type 2 inflammation"[Title/Abstract] OR “T2 inflammation"[Title/Abstract] OR “type 2 inflammatory"[Title/Abstract] OR “T2 inflammatory"[Title/Abstract] OR eosinophil*[Title/Abstract] OR “blood eosinophil count"[Title/Abstract] OR “absolute eosinophil count"[Title/Abstract] OR AEC[Title/Abstract] OR “eosinophilic asthma"[Title/Abstract] OR FeNO[Title/Abstract] OR “fractional exhaled nitric oxide"[Title/Abstract] OR IgE[Title/Abstract] OR “immunoglobulin E"[Title/Abstract] OR periostin[Title/Abstract]
#5	#1 AND #2 AND #3 AND #4

AUC, area under the receiver operating characteristic curve; FeNO, fractional exhaled nitric oxide; MeSH, Medical Subject Headings. The supplementary PubMed search was completed on 31 July 2026.

### 2.4 Study selection and data extraction

Two reviewers independently screened titles, abstracts, and full texts, with disagreements resolved by discussion or consultation with a third reviewer. Data extraction followed CHARMS (12) and covered study setting, population, sample size, event frequency, outcome definition, prediction horizon, candidate and final predictors, T2-biomarker definition and timing, missing-data handling, variable selection, algorithm, model presentation, validation, discrimination, calibration, classification measures, decision-curve analysis, and study limitations. When multiple models were reported, the model most relevant to the prespecified future-exacerbation outcome and the most independent validation result were preferentially extracted. For reports containing adult-only and mixed-age cohorts, only results evaluated in an adult cohort contributed to the main synthesis; results evaluated solely in a mixed-age cohort were described as contextual transportability evidence.

### 2.5 Risk of bias and applicability

Two reviewers independently assessed risk of bias and applicability using PROBAST (13). Risk of bias was evaluated across the participants, predictors, outcome, and analysis domains; applicability was assessed across the participants, predictors, and outcome domains. A domain was rated as low risk when all relevant signaling questions were answered as yes or probably yes, high risk when at least one critical item was answered as no or probably no, and unclear when reporting did not permit a judgment. The analysis domain considered effective sample size, predictor handling, missing data, class imbalance, overfitting, data leakage, calibration, and independence of validation.

### 2.6 Synthesis

A structured narrative synthesis was used because populations, outcomes, prediction horizons, biomarker definitions, algorithms, event prevalence, and performance metrics were substantially heterogeneous. Discrimination estimates were not pooled. Performance was interpreted jointly with calibration, validation, event prevalence, reporting transparency, and risk of bias rather than by ranking studies according to AUC alone (14).

## 3 Results

### 3.1 Study selection

The study-selection process is shown in Figure 1. After deduplication, title and abstract screening, and full-text assessment, eight eligible prediction-model reports were included in the qualitative synthesis (15–22).

**Figure 1:**
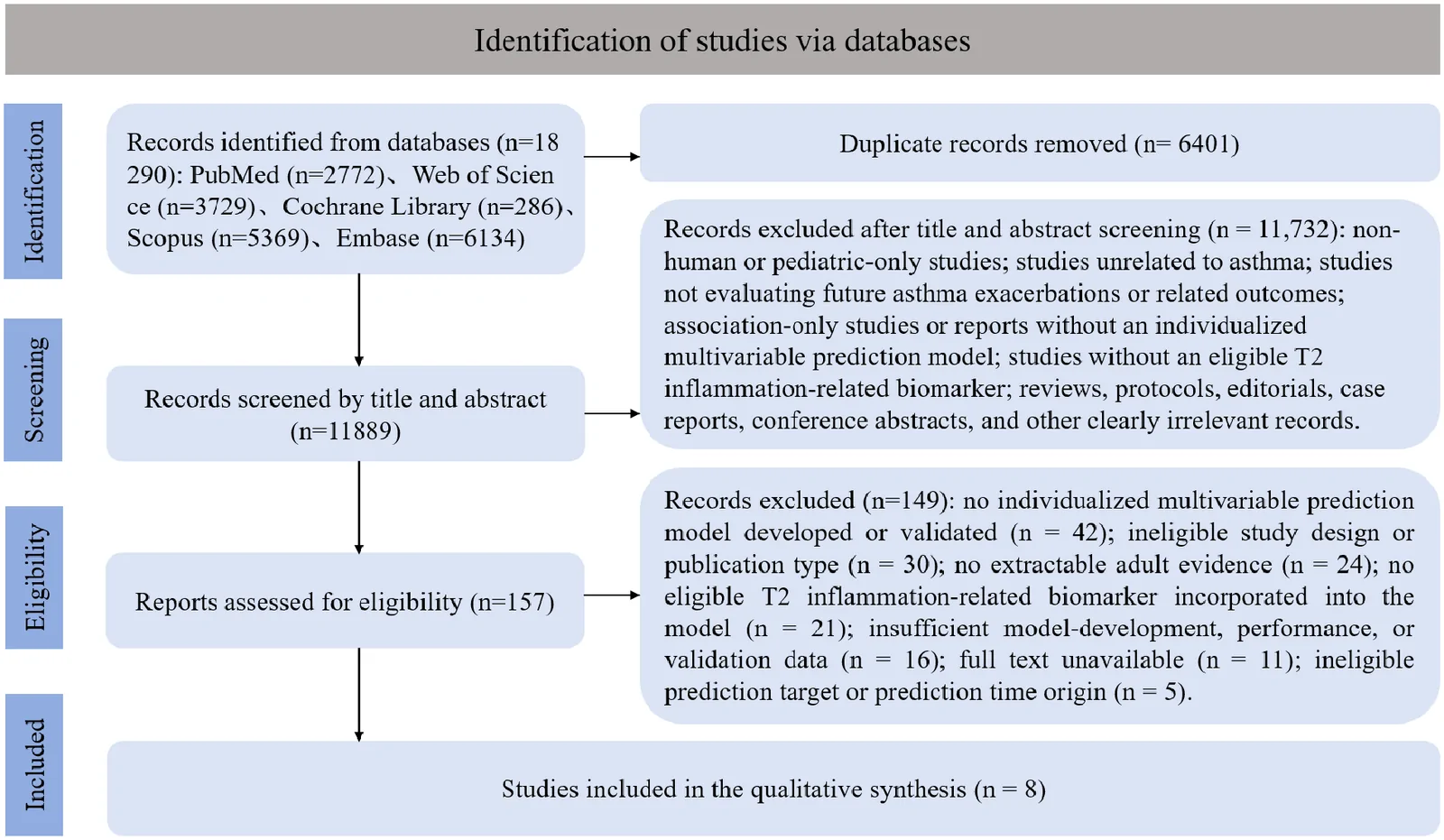
PRISMA 2020 flow diagram of study selection.

### 3.2 Characteristics of included studies

The eight included reports, published from 2021 to 2026, represented outpatient EHR cohorts, primary-care populations, biomarker-stratified clinical cohorts, multinational severe-asthma registries, a randomized-trial/real-world comparison, and adult biobank cohorts with targeted metabolomics. Eligible adult cohorts ranged from 540 to 1,331,934 participants, and prediction horizons ranged from 6 months to 5 years, most commonly 12 months. Eosinophil-related information appeared in all eight reports but was not retained in every final model. FeNO was included in the Yadav 2025 and Chen W 2026 registry models. Yadav 2026 compared 345 participants aged over 12 years from DREAM/MENSA placebo arms with 6,814 biologic-naive adults in ISAR; validation of the RCT-derived model in adult ISAR contributed to the main synthesis, whereas reverse validation in the mixed-age trial cohort was contextual. Yadav 2025, Yadav 2026, and Chen W 2026 partly used overlapping ISAR populations but addressed distinct questions and validation frameworks. Chen Y 2026 developed a 5-year model in 540 adults and replicated it in 823 adults. Table 3 summarizes study characteristics.

**Table 3:** Characteristics of included studies.

Study	Population/setting	Design and sample	Outcome/horizon	T2 biomarker role
Zein et al. 2021 (15)	Cleveland Clinic EHR; adults aged 18–80 years	Retrospective cohort; *n* = 60,302	OCS-treated exacerbation, ED visit, or hospitalization	Eosinophils and IgE as candidate inputs
Turcatel et al. 2025 (16)	US Optum Panther EHR; adults	Retrospective cohort; *n* = 1,331,934; events=16,279	6-month ED, urgent-care, or inpatient exacerbation	Eosinophil feature; SHAP importance
Tibble et al. 2025 (17)	Scottish linked care; adults receiving ICS	Retrospective cohort; *n* = 21,250	12-month attack: OCS, A&E/hospitalization, or death	BEC as final predictor
Pham et al. 2025 (18)	South Korea; BEC ≥300 vs. <150 cells/µL	Retrospective cohort; *n* = 669	12-month exacerbation	BEC as stratum and predictor
Yadav et al. 2026 (19)	DREAM/MENSA placebo arms and adult ISAR severe-asthma cohort	Secondary modeling study; mixed-age RCT *n* = 345; adult ISAR *n* = 6,814	0, 1, or ≥2 severe exacerbations over 365 days	BEC and total IgE as BN nodes; FeNO unavailable
Chen Y et al. 2026 (20)	MGBB-KAS and MGBB-LLF adult asthma biobank/EMR cohorts	Longitudinal discovery *n* = 540 and independent replication *n* = 823; complete-case model subsets	Any OCS-defined exacerbation during 5 years	IgE retained in best model; eosinophils in comparator models
Yadav et al. 2025 (21)	ISAR; biologic-naive severe asthma; 17 countries	Multinational cohort; *n* = 6,814	0, 1, or ≥2 severe exacerbations over 12 months	BEC and FeNO as Bayesian nodes
Chen W et al. 2026 (22)	ISAR/NOVEL; severe asthma; 30 countries/regions	Multinational cohort; *n* = 9,911	12-month risk of ≥1 or ≥2 severe exacerbations	BEC and FeNO as predictors

A&E, accident and emergency; AEC, absolute eosinophil count; BEC, blood eosinophil count; BN, Bayesian network; ED, emergency department; EHR/EMR, electronic health/medical record; ICS, inhaled corticosteroid; ISAR, International Severe Asthma Registry; OCS, oral corticosteroid. In Yadav 2026, validation in adult ISAR contributed to the main synthesis; reverse validation in the mixed-age RCT cohort was contextual.

### 3.3 Model development and validation

Modeling approaches included logistic regression, LightGBM and related machine-learning methods, XGBoost and deep-learning architectures, Lasso- or elastic-net-based selection, Bayesian networks, and a mixed-effects zero-inflated negative binomial model. Missing-data handling varied. Tibble et al. represented BEC as high, low, or missing; Yadav 2025 used random-forest imputation; Yadav 2026 used MissForest imputation, clinically informed discretization, and adaptive synthetic sampling; and Chen Y 2026 used complete-case clinical inputs and restricted ratio construction to metabolites with less than 30% missingness. Several EHR studies did not fully quantify the implications of selectively measured biomarkers. Validation ranged from same-source splits or cross-validation to temporal, country-stratified, internal-external, independent-cohort, and cross-source approaches (Table 4).

**Table 4:** Model development and validation characteristics.

Study	Algorithm	Missing data/feature handling	Validation	Output
Zein et al. 2021 (15)	Logistic regression; random forest; LightGBM	EHR data; variable laboratory availability	Same-system train/test split	Algorithm
Turcatel et al. 2025 (16)	XGBoost; LSTM; Transformer	Encoded EHR features	Same-source train/test split	Algorithm with SHAP
Tibble et al. 2025 (17)	Candidate ML; final logistic regression	BEC high/low/missing	Development/validation/test plus temporal assessment	Logistic equation
Pham et al. 2025 (18)	Lasso + logistic regression	Complete-/available-case analysis	70/30 internal split	Stratified models
Yadav et al. 2026 (19)	Hybrid expert-informed Bayesian networks	MissForest imputation; discretization; ADASYN class balancing	RCT-derived BN validated in adult ISAR; reverse mixed-age validation contextual	Bayesian pathway models
Chen Y et al. 2026 (20)	Elastic-net binomial regression; Cox models for single ratios	Ratios <30% missing; complete-case clinical inputs; final *n* not reported	Independent replication in MGBB-LLF	21-ratio derivation panel (16 ratios available in replication)
Yadav et al. 2025 (21)	Bayesian network/influence diagram	Random-forest imputation	10-fold and country-stratified validation	Bayesian network
Chen W et al. 2026 (22)	Mixed-effects zero-inflated negative binomial model	Routine predictors; country effects	Internal-external validation by country	RESA calculator

ADASYN, adaptive synthetic sampling; BEC, blood eosinophil count; BN, Bayesian network; EHR, electronic health record; LightGBM, light gradient-boosting machine; LSTM, long short-term memory; ML, machine learning; RESA, Risk of Exacerbation in Severe Asthma; SHAP, Shapley additive explanations; XGBoost, extreme gradient boosting.

### 3.4 Model performance and principal predictors

Discrimination varied substantially. Zein et al. reported LightGBM AUCs of approximately 0.71 for nonsevere exacerbations, 0.88 for emergency department visits, and 0.85 for hospitalization. Turcatel et al. reported an internally tested XGBoost AUC of 0.964 with an event prevalence of 1.2%. Tibble et al. reported an AUC of approximately 0.75, with a calibration slope of 1.02 and intercept of 0.004. Pham et al. reported AUCs of 0.84–0.85 in the high-BEC group and 0.93–0.94 in the low-BEC group, without calibration or external validation. In Yadav 2026, the RCT-derived Bayesian network achieved an AUC of 0.68 in the adult ISAR cohort; the reverse validation produced an AUC of 0.50 in the mixed-age RCT cohort and was treated as contextual evidence. The adult ISAR-derived network showed near-ideal calibration, with intercepts of 0.02 and −0.002 and slopes of 0.95 and 1.02 for at least one and at least two severe exacerbations. Chen Y 2026 reported AUCs of 0.901 and 0.893 in discovery and independent replication for a model combining sphingolipid-to-steroid ratios, serum IgE, race, and inhaled corticosteroid use. Yadav 2025 reported AUCs of 0.65 in cross-validation and 0.62 in country-stratified validation, with calibration estimates. Chen W 2026 reported pooled time-dependent AUCs of 0.63 for at least one and 0.68 for at least two severe exacerbations, with calibration and decision-curve analysis.

Across models, commonly represented clinical predictors included previous exacerbations, asthma control, reliever or systemic corticosteroid use, lung function, smoking, comorbidities, and treatment intensity. T2-related biomarkers generally supplemented rather than replaced these variables. Chen Y 2026 was the only report to provide an ablation-style T2-biomarker comparison: removing serum IgE reduced AUC from 0.901 to 0.793 in discovery and from 0.893 to 0.724 in replication. Because this was not a prespecified comparison with a standard clinical base model and used complete cases, it should not be interpreted as definitive incremental value. No study directly isolated the incremental value of BEC or FeNO beyond a prespecified clinical model (Table 5).

**Table 5:** Performance and interpretation of included models.

Study	Main discrimination	Calibration/utility	Key strength/limitation
Zein et al. 2021 (15)	LightGBM AUC 0.71 (nonsevere), 0.88 (ED), 0.85 (hospitalization)	Not fully reported	Single system; biomarker increment unclear
Turcatel et al. 2025 (16)	XGBoost AUC 0.964	No external validation; event rate 1.2%	High internal AUC; limited transportability
Tibble et al. 2025 (17)	AUC about 0.75	Slope 1.02; intercept 0.004; PPV 36.2%	Calibration reported
Pham et al. 2025 (18)	High-BEC AUC 0.84/0.85; low-BEC 0.93/0.94	No calibration; internal split	Few events; no external validation
Yadav et al. 2026 (19)	RCT-BN to adult ISAR AUC 0.68; reverse mixed-age validation AUC 0.50	Adult ISAR intercept 0.02/-0.002; slope 0.95/1.02	Cross-source validation; mixed-age RCT comparator
Chen Y et al. 2026 (20)	AUC 0.901 discovery; 0.893 replication	No calibration or DCA; without IgE AUC 0.793/0.724	Independent replication; OCS proxy and complete-case analysis
Yadav et al. 2025 (21)	AUC 0.65 (cross-validation); 0.62 (country validation)	Intercept 0.130/-0.018; slope 0.804/0.949	Country-stratified validation
Chen W et al. 2026 (22)	AUC 0.63 (≥1); 0.68 (≥2 severe exacerbations)	Calibration close to ideal; net benefit reported	Internal-external validation

AUC, area under the receiver operating characteristic curve; BEC, blood eosinophil count; BN, Bayesian network; DCA, decision-curve analysis; ED, emergency department; IgE, immunoglobulin E; ISAR, International Severe Asthma Registry; OCS, oral corticosteroid; PPV, positive predictive value; RCT, randomized controlled trial. Estimates are not directly comparable because populations, outcomes, horizons, and validation designs differed.

### 3.5 Application of T2 inflammation-related biomarkers

Eosinophil-related variables were the most common T2-biomarker input, serving as candidate EHR features in Zein et al., encoded SHAP-interpreted features in Turcatel et al., a categorized predictor in Tibble et al., a stratification variable and predictor in Pham et al., Bayesian-network nodes in Yadav 2025 and Yadav 2026, direct predictors in Chen W 2026, and comparator-model variables in Chen Y 2026. FeNO was included in the Yadav 2025 and Chen W 2026 severe-asthma registry models. Total serum IgE was included as a candidate input or network node in several reports and retained in the best-performing Chen Y 2026 model. In Yadav 2026, the learned network contained an arc from IgE to BEC and from BEC to future severe exacerbations; these probabilistic dependencies do not establish causality. FeNO was excluded because it was unavailable in one trial. Periostin was not incorporated in an eligible model.

The Turcatel report requires cautious interpretation because its methods and SHAP analyses describe eosinophil laboratory encoding, whereas the discussion notes incomplete availability of routinely used T2 biomarkers, including BEC and FeNO. Chen Y 2026 also warrants caution: higher discrimination after adding serum IgE was observed in an ablation comparison, but IgE was missing in 67.2% of the discovery cohort and 56.9% of the replication cohort, and the final analysis used complete cases. These issues were treated as concerns about transparency, selection, and applicability rather than definitive evidence of clinical biomarker utility (Table 6).

**Table 6:** Role of T2 biomarkers in eligible prediction models.

Biomarker	Eligible studies	Role	Evidence limitation
BEC/AEC/eosinophil feature	All eight reports	Candidate, stratum, node, final predictor, or comparator-model input	Not retained in every final model; variable timing and missingness
FeNO	Yadav 2025; Chen W 2026	Predictor in severe-asthma models	Severe-asthma evidence only; incremental value unclear
IgE	Zein 2021; Yadav 2025; Yadav 2026; Chen Y 2026; Chen W 2026	Candidate input, network node, or retained predictor	Frequent missingness; ablation comparison in one report
Periostin	No eligible model	-	Association-only evidence

AEC, absolute eosinophil count; BEC, blood eosinophil count; FeNO, fractional exhaled nitric oxide; IgE, immunoglobulin E; T2, type 2. Inclusion in this table does not imply independent incremental value or clinical readiness.

### 3.6 Risk of bias and applicability

Risk of bias was concentrated in the analysis domain. Common concerns included same-source internal validation, incomplete calibration reporting, sparse or selectively measured biomarkers, unclear missing-data or class-imbalance handling, complete-case analysis, flexible feature selection, and limited assessment of incremental biomarker value. Zein, Turcatel, Tibble, Pham, Yadav 2026, and Chen Y 2026 were rated at high overall risk of bias. For Yadav 2026, concerns included the mixed-age RCT comparator, low event information in the RCT cohort, extensive imputation and discretization, and synthetic oversampling. For Chen Y 2026, concerns included complete-case analysis, an OCS-prescription proxy for exacerbations, absent calibration, and incomplete reporting of final model sample sizes. Yadav 2025 had comparatively complete validation and calibration reporting, while Chen W 2026 showed the most complete validation profile, including internal-external cross-validation, calibration, and clinical net benefit. Applicability was generally acceptable for the intended adult populations, although severe-asthma registry and metabolomics models may not transport directly to unselected primary care (Table 7).

**Table 7:** PROBAST risk-of-bias and applicability assessment.

Study	Participants	Predictors	Outcome	Analysis	Overall ROB	Applicability
Zein 2021	Low	Low	Low	High	High	Low concern
Turcatel 2025	Low	High	Low	High	High	Some concern
Tibble 2025	Low	Low	Low	High	High	Low concern
Pham 2025	Low	Low	Low	High	High	Low concern
Yadav 2026	High	Low	Low	High	High	Some concern
Chen Y 2026	Low	Low	High	High	High	Some concern
Yadav 2025	Low	Low	Low	Low	Low	Some concern
Chen W 2026	Low	Low	Low	Low	Low	Some concern

RoB, risk of bias. Overall RoB reflects the four PROBAST domains. For Yadav 2026, validation in adult ISAR contributed to the main synthesis; the mixed-age RCT cohort informed contextual transportability and the report-level participant judgment.

## 4 Discussion

This systematic review identified eight prognostic-model reports with extractable adult evidence that incorporated T2-related biomarkers. The main finding is not that BEC, FeNO, or IgE constitutes a validated stand-alone solution, but that these biomarkers are increasingly embedded in broader models that also use previous exacerbations, asthma control, medication use, lung function, comorbidities, and, in one report, metabolomic signatures. The evidence remains small, heterogeneous, and methodologically uneven; apparent performance therefore requires interpretation alongside validation strategy, missing-data handling, and intended setting.

A key methodological feature of this review is the separation of prognostic association from individualized prediction. Association estimates alone do not constitute a deployable model; an eligible model had to generate an individual risk output and report performance or validation. This distinction reduces the risk of treating population-level associations as clinically validated tools.

Lee et al. was retained as contextual evidence but excluded from the qualitative synthesis because prediction began after an index exacerbation and targeted a 12-month post-index relapse composite, whereas the prespecified review setting was baseline assessment outside an acute episode (23). The brief report also provided limited detail on the algorithm, missing-data handling, calibration, and model presentation. It nevertheless illustrates that post-exacerbation relapse prediction is a related but distinct task. Prototype and *post hoc* tools were eligible only when adult participant-level data generated individualized risk estimates with performance evaluation; aggregate biomarker strata and association-only analyses remained contextual evidence.

The present review differs from previous reviews in its analytic focus. Ma and Tibble evaluated primary-care asthma-attack models, while Liu et al. assessed broader adult exacerbation models and their risk of bias (8, 9). Requiring explicit incorporation of a T2-related biomarker allowed assessment of how the biomarker was measured, handled when missing, modeled, separated from established clinical predictors, and evaluated for calibration or clinical utility. An earlier review with external validation likewise found that performance varied across populations and outcome definitions, underscoring the importance of transportability (24).

Model performance should be assessed across discrimination, calibration, overall performance, and clinical utility rather than by AUC alone (25). Repeated measurements in severe asthma have demonstrated frequent crossing of clinically used BEC and FeNO thresholds, reinforcing the importance of documenting measurement timing and considering repeat testing (26). In mild asthma, blood eosinophils, but not FeNO, modified the relative benefit of maintenance inhaled corticosteroid therapy, illustrating that prognostic and treatment-predictive roles are population dependent (27). A prospective real-world severe-asthma study found no prognostic value for periostin, whereas concurrently elevated blood eosinophils and FeNO were potentially associated with higher exacerbation rates (28).

The contrast between discrimination and validation was important. Some internally evaluated models reported very high AUCs, whereas models subjected to country-based, internal-external, independent-cohort, or cross-source validation showed more modest or variable discrimination. High apparent discrimination can reflect case mix, low event prevalence, flexible algorithms, or overfitting and does not establish transportability (29). Calibration is equally important because clinical decisions depend on agreement between predicted and observed risks (30). Accordingly, the lower AUCs reported by Yadav 2025, Yadav 2026, and Chen W 2026 should not be interpreted automatically as weaker evidence when validation or calibration was more complete (19, 21, 22). Conversely, Chen Y 2026 reported encouraging independent-cohort AUCs, but absent calibration and complete-case analysis limit implementation claims (20).

The two newly included reports broaden the evidence beyond conventional BEC- and FeNO-centered models. Yadav 2026 found a shared downstream network structure containing IgE-BEC and BEC-future-exacerbation arcs, alongside a direct pathway from previous to future severe exacerbations (19). The RCT-derived network showed better cross-source discrimination in adult ISAR than the adult ISAR-derived network did in the mixed-age RCT cohort, but this asymmetry may reflect differences in age, case mix, event frequency, and data environment. Chen Y 2026 provided the only ablation-style comparison of a T2 biomarker: adding IgE to the ratio-based model was associated with higher AUCs in discovery and replication (20). This finding is supportive rather than practice-changing because IgE was frequently missing, complete cases were analyzed, exacerbations were proxied by OCS prescriptions, and calibration and decision-curve analysis were not reported.

The biological and analytical behavior of T2 biomarkers creates additional challenges for model development. Longitudinal studies have shown that clinically used T2 biomarkers may vary over time within individual patients, even when cohort-level patterns appear relatively stable (26, 31). FeNO may add prognostic information to BEC and prior exacerbation history in selected moderate-to-severe asthma populations, but its contribution is conditional on population characteristics, background therapy, and the timing of measurement (32). Models based on a single opportunistic laboratory value may therefore misclassify patients whose biomarker level is transiently suppressed by corticosteroids or altered by adherence, infection, smoking, or allergen exposure. The new metabolomics evidence adds a related problem: even when a biomarker improves discrimination, selective measurement and complete-case analysis may create a model population that differs materially from the intended clinical population.

BEC and FeNO represent related but non-equivalent aspects of T2 biology: BEC reflects systemic eosinophilic burden, whereas FeNO more directly reflects IL-4/IL-13-associated airway signaling. Observational evidence suggests that concurrent elevation identifies greater exacerbation burden than either marker alone (33). Total serum IgE reflects another component of T2 biology, but its pathway role and incremental predictive value are less consistently studied. Yadav 2026 and Chen Y 2026 suggest that IgE may add information in combination with eosinophilic or metabolic variables rather than as a stand-alone marker (19, 20). FeNO interpretation nevertheless requires standardized measurement and clinically meaningful cut points, and thresholds developed for diagnosis or treatment selection cannot be assumed to perform equivalently in prognostic models (34). Future models should examine continuous effects, nonlinear relationships, repeated measurements, and interactions with corticosteroid exposure rather than relying only on fixed binary thresholds.

The distinction between prognostic usefulness and treatment usefulness must also be maintained. A biomarker may improve risk estimation without demonstrating that biomarker-guided action improves outcomes. In severe asthma, a composite T2-biomarker strategy has been evaluated as a means of adjusting corticosteroid treatment, illustrating the additional evidentiary step required to move from risk stratification to management decisions (35). Prospective impact studies are ultimately needed to establish whether use of a prediction model changes clinician behavior, treatment allocation, or patient outcomes.

Transportability is likely to vary by setting. Large EHR models may capture broad real-world populations and complex interactions, but laboratory measurements are often selective, coding practices vary, and event definitions depend on local care. Severe-asthma registries provide richer biomarker data but represent selected specialist populations, while metabolomics cohorts require assays and preprocessing not routinely available. The Zein and Turcatel models therefore cannot be assumed to transfer to multinational specialist registries, and the Yadav and Chen models may require recalibration before primary-care use (15, 16, 19–22). The three ISAR-based reports also share participants and should not be interpreted as independent replications. External validation should assess overall performance and subgroups defined by severity, biologic use, smoking, comorbidity, and health-care system.

Sample size and effective event information warrant greater attention. Several models considered many candidate predictors relative to the number of exacerbation events, increasing the risk of unstable variable selection and optimistic performance. Modern sample-size guidance recommends basing development requirements on the anticipated model fit, number of predictor parameters, outcome prevalence, and acceptable optimism rather than relying on a universal events-per-variable rule (36). External validation likewise requires enough events and non-events to estimate discrimination, calibration, and overall risk with adequate precision (37). These requirements are especially relevant when biomarker measurements are available only in a subset of the cohort, when complete-case analysis sharply reduces the effective sample, or when separate models are developed within biomarker strata.

Clinical utility should be assessed explicitly rather than inferred from AUC. Decision-curve analysis quantifies whether model-guided action provides greater net benefit than treating all or no patients across plausible risk thresholds (38). In addition, continuous predictors such as BEC, FeNO, and IgE should generally be retained on their original scale, with nonlinear terms considered where appropriate, because arbitrary dichotomization discards information and may reduce transportability (39). For metabolomic panels, the intended assay, preprocessing pipeline, coefficients, and risk calculation must also be fully specified. Accessible equations, point scores, electronic calculators, or interoperable clinical decision-support tools are necessary if a model is intended for implementation.

Future studies should follow TRIPOD + AI irrespective of whether regression or machine-learning methods are used (40). Investigators should prespecify the intended moment of use, prediction horizon, outcome definition, biomarker measurement window, handling of corticosteroid exposure, missing-data strategy, and the clinical comparator model. Development should use robust internal validation, followed by geographic or health-system external validation, recalibration when necessary, and prospective impact evaluation. Most importantly, studies should report the incremental contribution of each T2 biomarker beyond established clinical predictors using changes in calibration, discrimination, and net benefit, rather than treating biomarker retention in a final algorithm as sufficient evidence of value.

## 5 Strengths and limitations

Strengths of this review include a prediction-model-specific eligibility framework, CHARMS-guided extraction, and PROBAST assessment; several limitations remain. (1) Only eight eligible reports were identified, and heterogeneity in populations, outcomes, horizons, biomarker measurements, modeling methods, and performance metrics precluded quantitative synthesis. (2) Model equations, predictor definitions, biomarker timing, missing-data handling, class imbalance, calibration, and final analytic sample sizes were incompletely reported in several studies, limiting reproducibility. (3) Yadav 2025, Yadav 2026, and Chen W 2026 partly used overlapping ISAR populations, so participant samples were not fully independent. (4) The supplementary update was restricted to PubMed, and the baseline-risk boundary was operationally clarified after registration; both may have affected study identification or borderline eligibility judgments. (5) Individual participant data were unavailable, preventing subgroup analyses, independent recalibration, and direct comparison of the incremental value of BEC, FeNO, and IgE.

Future research should define the intended prediction time origin and horizon clearly, standardize exacerbation outcomes, use repeated or appropriately timed T2-biomarker measurements, and transparently address corticosteroid exposure and missing data. Model development should be supported by adequate event numbers and robust internal validation, followed by independent geographic or health-system validation (36, 37). Studies should also report calibration, decision-curve analysis, accessible model equations or electronic tools, and prospective clinical-impact evaluations (38, 40). Most importantly, models with and without individual T2 biomarkers should be compared directly to quantify their incremental contributions to discrimination, calibration, and clinical net benefit.

## 6 Conclusion

T2-related biomarkers are increasingly incorporated into adult asthma exacerbation-risk models and may add information beyond established clinical predictors. The newly included evidence indicates that IgE may contribute in specific Bayesian-network and metabolomics contexts, but this requires independent confirmation with complete biomarker ascertainment and calibration. Current evidence does not support naming BEC, FeNO, or IgE as the most useful predictor or adopting an existing model in routine care. Progress will require adequate sample size, standardized biomarker measurement, transparent missing-data handling, formal incremental-value assessment, external validation, calibration, clinical-utility analysis, and prospective impact evaluation.

## Data Availability

The original contributions presented in the study are included in the article/Supplementary Material, further inquiries can be directed to the corresponding author.
