# Supplementary material for: Application of type 2 inflammation-related biomarkers in risk prediction models for acute exacerbations in adult asthma: a systematic review
Source: Front Med (Lausanne). 2026 Sep 18;13:1900214. doi: 10.3389/fmed.2026.1900214 (PMC13630933; doi:10.3389/fmed.2026.1900214)
Supplement: Supplementary file 1 [file Table1.docx]

Supplementary Material

[Supplementary Table 1: Search Results	2](#_Toc231488784)

# Supplementary Table 1: Search Results

The search strategies for each database were implemented on July 31, 2026. After appropriate adaptation and adjustment, the strategies can also be applied to retrieve relevant studies in other electronic databases. PubMed (2772)

| **Search number** | **Query** | **Search Details** | **Results** |
| --- | --- | --- | --- |
| **13** | (((("Asthma"[Mesh]) OR (asthma[Title/Abstract] OR bronchial asthma[Title/Abstract] OR asthmatic[Title/Abstract])) AND (("Disease Progression"[Mesh]) OR (exacerbat*[Title/Abstract] OR "acute attack"[Title/Abstract] OR "acute attacks"[Title/Abstract] OR "asthma attack"[Title/Abstract] OR "asthma attacks"[Title/Abstract] OR worsen*[Title/Abstract] OR hospitali*[Title/Abstract] OR "emergency department"[Title/Abstract] OR "emergency departments"[Title/Abstract] OR "emergency visit"[Title/Abstract] OR "emergency visits"[Title/Abstract] OR "oral corticosteroid"[Title/Abstract] OR "oral corticosteroids"[Title/Abstract] OR "systemic corticosteroid"[Title/Abstract] OR "systemic corticosteroids"[Title/Abstract] OR glucocorticoid*[Title/Abstract]))) AND (("Models, Statistical"[Mesh]) OR (predict*[Title/Abstract] OR prognos*[Title/Abstract] OR model*[Title/Abstract] OR "risk prediction"[Title/Abstract] OR "risk assessment"[Title/Abstract] OR "prediction model"[Title/Abstract] OR "prediction models"[Title/Abstract] OR "prognostic model"[Title/Abstract] OR "prognostic models"[Title/Abstract] OR "risk model"[Title/Abstract] OR "risk models"[Title/Abstract] OR "risk score"[Title/Abstract] OR "risk scores"[Title/Abstract] OR "risk index"[Title/Abstract] OR "risk indices"[Title/Abstract] OR "risk calculator"[Title/Abstract] OR "risk calculators"[Title/Abstract] OR nomogram*[Title/Abstract] OR "machine learning"[Title/Abstract] OR "artificial intelligence"[Title/Abstract] OR "deep learning"[Title/Abstract] OR "logistic regression"[Title/Abstract] OR "Cox regression"[Title/Abstract] OR "proportional hazards"[Title/Abstract] OR Lasso[Title/Abstract] OR "random forest"[Title/Abstract] OR "gradient boosting"[Title/Abstract] OR "Bayesian network"[Title/Abstract] OR "Bayesian networks"[Title/Abstract] OR validation[Title/Abstract] OR calibration[Title/Abstract] OR discrimination[Title/Abstract] OR AUC[Title/Abstract] OR "decision curve"[Title/Abstract]))) AND (("Eosinophils"[Mesh]) OR ("type 2 inflammation"[Title/Abstract] OR "T2 inflammation"[Title/Abstract] OR "type 2 inflammatory"[Title/Abstract] OR "T2 inflammatory"[Title/Abstract] OR eosinophil*[Title/Abstract] OR "blood eosinophil count"[Title/Abstract] OR "absolute eosinophil count"[Title/Abstract] OR AEC[Title/Abstract] OR "eosinophilic asthma"[Title/Abstract] OR FeNO[Title/Abstract] OR "fractional exhaled nitric oxide"[Title/Abstract] OR IgE[Title/Abstract] OR "immunoglobulin E"[Title/Abstract] OR periostin[Title/Abstract])) | ("Asthma"[MeSH Terms] OR ("Asthma"[Title/Abstract] OR "bronchial asthma"[Title/Abstract] OR "asthmatic"[Title/Abstract])) AND ("Disease Progression"[MeSH Terms] OR ("exacerbat*"[Title/Abstract] OR "acute attack"[Title/Abstract] OR "acute attacks"[Title/Abstract] OR "asthma attack"[Title/Abstract] OR "asthma attacks"[Title/Abstract] OR "worsen*"[Title/Abstract] OR "hospitali*"[Title/Abstract] OR "emergency department"[Title/Abstract] OR "emergency departments"[Title/Abstract] OR "emergency visit"[Title/Abstract] OR "emergency visits"[Title/Abstract] OR "oral corticosteroid"[Title/Abstract] OR "oral corticosteroids"[Title/Abstract] OR "systemic corticosteroid"[Title/Abstract] OR "systemic corticosteroids"[Title/Abstract] OR "glucocorticoid*"[Title/Abstract])) AND ("models, statistical"[MeSH Terms] OR ("predict*"[Title/Abstract] OR "prognos*"[Title/Abstract] OR "model*"[Title/Abstract] OR "risk prediction"[Title/Abstract] OR "risk assessment"[Title/Abstract] OR "prediction model"[Title/Abstract] OR "prediction models"[Title/Abstract] OR "prognostic model"[Title/Abstract] OR "prognostic models"[Title/Abstract] OR "risk model"[Title/Abstract] OR "risk models"[Title/Abstract] OR "risk score"[Title/Abstract] OR "risk scores"[Title/Abstract] OR "risk index"[Title/Abstract] OR "risk indices"[Title/Abstract] OR "risk calculator"[Title/Abstract] OR "risk calculators"[Title/Abstract] OR "nomogram*"[Title/Abstract] OR "machine learning"[Title/Abstract] OR "artificial intelligence"[Title/Abstract] OR "deep learning"[Title/Abstract] OR "logistic regression"[Title/Abstract] OR "Cox regression"[Title/Abstract] OR "proportional hazards"[Title/Abstract] OR "Lasso"[Title/Abstract] OR "random forest"[Title/Abstract] OR "gradient boosting"[Title/Abstract] OR "Bayesian network"[Title/Abstract] OR "Bayesian networks"[Title/Abstract] OR "validation"[Title/Abstract] OR "calibration"[Title/Abstract] OR "discrimination"[Title/Abstract] OR "AUC"[Title/Abstract] OR "decision curve"[Title/Abstract])) AND ("Eosinophils"[MeSH Terms] OR ("type 2 inflammation"[Title/Abstract] OR "T2 inflammation"[Title/Abstract] OR "type 2 inflammatory"[Title/Abstract] OR "T2 inflammatory"[Title/Abstract] OR "eosinophil*"[Title/Abstract] OR "blood eosinophil count"[Title/Abstract] OR "absolute eosinophil count"[Title/Abstract] OR "AEC"[Title/Abstract] OR "eosinophilic asthma"[Title/Abstract] OR "FeNO"[Title/Abstract] OR "fractional exhaled nitric oxide"[Title/Abstract] OR "IgE"[Title/Abstract] OR "immunoglobulin E"[Title/Abstract] OR "periostin"[Title/Abstract])) | 2772 |
| **12** | ("Eosinophils"[Mesh]) OR ("type 2 inflammation"[Title/Abstract] OR "T2 inflammation"[Title/Abstract] OR "type 2 inflammatory"[Title/Abstract] OR "T2 inflammatory"[Title/Abstract] OR eosinophil*[Title/Abstract] OR "blood eosinophil count"[Title/Abstract] OR "absolute eosinophil count"[Title/Abstract] OR AEC[Title/Abstract] OR "eosinophilic asthma"[Title/Abstract] OR FeNO[Title/Abstract] OR "fractional exhaled nitric oxide"[Title/Abstract] OR IgE[Title/Abstract] OR "immunoglobulin E"[Title/Abstract] OR periostin[Title/Abstract]) | "Eosinophils"[MeSH Terms] OR "type 2 inflammation"[Title/Abstract] OR "T2 inflammation"[Title/Abstract] OR "type 2 inflammatory"[Title/Abstract] OR "T2 inflammatory"[Title/Abstract] OR "eosinophil*"[Title/Abstract] OR "blood eosinophil count"[Title/Abstract] OR "absolute eosinophil count"[Title/Abstract] OR "AEC"[Title/Abstract] OR "eosinophilic asthma"[Title/Abstract] OR "FeNO"[Title/Abstract] OR "fractional exhaled nitric oxide"[Title/Abstract] OR "IgE"[Title/Abstract] OR "immunoglobulin E"[Title/Abstract] OR "periostin"[Title/Abstract] | 175,168 |
| **11** | "type 2 inflammation"[Title/Abstract] OR "T2 inflammation"[Title/Abstract] OR "type 2 inflammatory"[Title/Abstract] OR "T2 inflammatory"[Title/Abstract] OR eosinophil*[Title/Abstract] OR "blood eosinophil count"[Title/Abstract] OR "absolute eosinophil count"[Title/Abstract] OR AEC[Title/Abstract] OR "eosinophilic asthma"[Title/Abstract] OR FeNO[Title/Abstract] OR "fractional exhaled nitric oxide"[Title/Abstract] OR IgE[Title/Abstract] OR "immunoglobulin E"[Title/Abstract] OR periostin[Title/Abstract] | "type 2 inflammation"[Title/Abstract] OR "T2 inflammation"[Title/Abstract] OR "type 2 inflammatory"[Title/Abstract] OR "T2 inflammatory"[Title/Abstract] OR "eosinophil*"[Title/Abstract] OR "blood eosinophil count"[Title/Abstract] OR "absolute eosinophil count"[Title/Abstract] OR "AEC"[Title/Abstract] OR "eosinophilic asthma"[Title/Abstract] OR "FeNO"[Title/Abstract] OR "fractional exhaled nitric oxide"[Title/Abstract] OR "IgE"[Title/Abstract] OR "immunoglobulin E"[Title/Abstract] OR "periostin"[Title/Abstract] | 171117 |
| **10** | "Eosinophils"[Mesh] | "Eosinophils"[MeSH Terms] | 27,531 |
| **9** | ("Models, Statistical"[Mesh]) OR (predict*[Title/Abstract] OR prognos*[Title/Abstract] OR model*[Title/Abstract] OR "risk prediction"[Title/Abstract] OR "risk assessment"[Title/Abstract] OR "prediction model"[Title/Abstract] OR "prediction models"[Title/Abstract] OR "prognostic model"[Title/Abstract] OR "prognostic models"[Title/Abstract] OR "risk model"[Title/Abstract] OR "risk models"[Title/Abstract] OR "risk score"[Title/Abstract] OR "risk scores"[Title/Abstract] OR "risk index"[Title/Abstract] OR "risk indices"[Title/Abstract] OR "risk calculator"[Title/Abstract] OR "risk calculators"[Title/Abstract] OR nomogram*[Title/Abstract] OR "machine learning"[Title/Abstract] OR "artificial intelligence"[Title/Abstract] OR "deep learning"[Title/Abstract] OR "logistic regression"[Title/Abstract] OR "Cox regression"[Title/Abstract] OR "proportional hazards"[Title/Abstract] OR Lasso[Title/Abstract] OR "random forest"[Title/Abstract] OR "gradient boosting"[Title/Abstract] OR "Bayesian network"[Title/Abstract] OR "Bayesian networks"[Title/Abstract] OR validation[Title/Abstract] OR calibration[Title/Abstract] OR discrimination[Title/Abstract] OR AUC[Title/Abstract] OR "decision curve"[Title/Abstract]) | "models, statistical"[MeSH Terms] OR "predict*"[Title/Abstract] OR "prognos*"[Title/Abstract] OR "model*"[Title/Abstract] OR "risk prediction"[Title/Abstract] OR "risk assessment"[Title/Abstract] OR "prediction model"[Title/Abstract] OR "prediction models"[Title/Abstract] OR "prognostic model"[Title/Abstract] OR "prognostic models"[Title/Abstract] OR "risk model"[Title/Abstract] OR "risk models"[Title/Abstract] OR "risk score"[Title/Abstract] OR "risk scores"[Title/Abstract] OR "risk index"[Title/Abstract] OR "risk indices"[Title/Abstract] OR "risk calculator"[Title/Abstract] OR "risk calculators"[Title/Abstract] OR "nomogram*"[Title/Abstract] OR "machine learning"[Title/Abstract] OR "artificial intelligence"[Title/Abstract] OR "deep learning"[Title/Abstract] OR "logistic regression"[Title/Abstract] OR "Cox regression"[Title/Abstract] OR "proportional hazards"[Title/Abstract] OR "Lasso"[Title/Abstract] OR "random forest"[Title/Abstract] OR "gradient boosting"[Title/Abstract] OR "Bayesian network"[Title/Abstract] OR "Bayesian networks"[Title/Abstract] OR "validation"[Title/Abstract] OR "calibration"[Title/Abstract] OR "discrimination"[Title/Abstract] OR "AUC"[Title/Abstract] OR "decision curve"[Title/Abstract] | 8,219,410 |
| **8** | predict*[Title/Abstract] OR prognos*[Title/Abstract] OR model*[Title/Abstract] OR "risk prediction"[Title/Abstract] OR "risk assessment"[Title/Abstract] OR "prediction model"[Title/Abstract] OR "prediction models"[Title/Abstract] OR "prognostic model"[Title/Abstract] OR "prognostic models"[Title/Abstract] OR "risk model"[Title/Abstract] OR "risk models"[Title/Abstract] OR "risk score"[Title/Abstract] OR "risk scores"[Title/Abstract] OR "risk index"[Title/Abstract] OR "risk indices"[Title/Abstract] OR "risk calculator"[Title/Abstract] OR "risk calculators"[Title/Abstract] OR nomogram*[Title/Abstract] OR "machine learning"[Title/Abstract] OR "artificial intelligence"[Title/Abstract] OR "deep learning"[Title/Abstract] OR "logistic regression"[Title/Abstract] OR "Cox regression"[Title/Abstract] OR "proportional hazards"[Title/Abstract] OR Lasso[Title/Abstract] OR "random forest"[Title/Abstract] OR "gradient boosting"[Title/Abstract] OR "Bayesian network"[Title/Abstract] OR "Bayesian networks"[Title/Abstract] OR validation[Title/Abstract] OR calibration[Title/Abstract] OR discrimination[Title/Abstract] OR AUC[Title/Abstract] OR "decision curve"[Title/Abstract] | "predict*"[Title/Abstract] OR "prognos*"[Title/Abstract] OR "model*"[Title/Abstract] OR "risk prediction"[Title/Abstract] OR "risk assessment"[Title/Abstract] OR "prediction model"[Title/Abstract] OR "prediction models"[Title/Abstract] OR "prognostic model"[Title/Abstract] OR "prognostic models"[Title/Abstract] OR "risk model"[Title/Abstract] OR "risk models"[Title/Abstract] OR "risk score"[Title/Abstract] OR "risk scores"[Title/Abstract] OR "risk index"[Title/Abstract] OR "risk indices"[Title/Abstract] OR "risk calculator"[Title/Abstract] OR "risk calculators"[Title/Abstract] OR "nomogram*"[Title/Abstract] OR "machine learning"[Title/Abstract] OR "artificial intelligence"[Title/Abstract] OR "deep learning"[Title/Abstract] OR "logistic regression"[Title/Abstract] OR "Cox regression"[Title/Abstract] OR "proportional hazards"[Title/Abstract] OR "Lasso"[Title/Abstract] OR "random forest"[Title/Abstract] OR "gradient boosting"[Title/Abstract] OR "Bayesian network"[Title/Abstract] OR "Bayesian networks"[Title/Abstract] OR "validation"[Title/Abstract] OR "calibration"[Title/Abstract] OR "discrimination"[Title/Abstract] OR "AUC"[Title/Abstract] OR "decision curve"[Title/Abstract] | 8,081,395 |
| **7** | "Models, Statistical"[Mesh] | "models, statistical"[MeSH Terms] | 490,722 |
| **6** | ("Disease Progression"[Mesh]) OR (exacerbat*[Title/Abstract] OR "acute attack"[Title/Abstract] OR "acute attacks"[Title/Abstract] OR "asthma attack"[Title/Abstract] OR "asthma attacks"[Title/Abstract] OR worsen*[Title/Abstract] OR hospitali*[Title/Abstract] OR "emergency department"[Title/Abstract] OR "emergency departments"[Title/Abstract] OR "emergency visit"[Title/Abstract] OR "emergency visits"[Title/Abstract] OR "oral corticosteroid"[Title/Abstract] OR "oral corticosteroids"[Title/Abstract] OR "systemic corticosteroid"[Title/Abstract] OR "systemic corticosteroids"[Title/Abstract] OR glucocorticoid*[Title/Abstract]) | "Disease Progression"[MeSH Terms] OR "exacerbat*"[Title/Abstract] OR "acute attack"[Title/Abstract] OR "acute attacks"[Title/Abstract] OR "asthma attack"[Title/Abstract] OR "asthma attacks"[Title/Abstract] OR "worsen*"[Title/Abstract] OR "hospitali*"[Title/Abstract] OR "emergency department"[Title/Abstract] OR "emergency departments"[Title/Abstract] OR "emergency visit"[Title/Abstract] OR "emergency visits"[Title/Abstract] OR "oral corticosteroid"[Title/Abstract] OR "oral corticosteroids"[Title/Abstract] OR "systemic corticosteroid"[Title/Abstract] OR "systemic corticosteroids"[Title/Abstract] OR "glucocorticoid*"[Title/Abstract] | 1221639 |
| **5** | exacerbat*[Title/Abstract] OR "acute attack"[Title/Abstract] OR "acute attacks"[Title/Abstract] OR "asthma attack"[Title/Abstract] OR "asthma attacks"[Title/Abstract] OR worsen*[Title/Abstract] OR hospitali*[Title/Abstract] OR "emergency department"[Title/Abstract] OR "emergency departments"[Title/Abstract] OR "emergency visit"[Title/Abstract] OR "emergency visits"[Title/Abstract] OR "oral corticosteroid"[Title/Abstract] OR "oral corticosteroids"[Title/Abstract] OR "systemic corticosteroid"[Title/Abstract] OR "systemic corticosteroids"[Title/Abstract] OR glucocorticoid*[Title/Abstract] | "exacerbat*"[Title/Abstract] OR "acute attack"[Title/Abstract] OR "acute attacks"[Title/Abstract] OR "asthma attack"[Title/Abstract] OR "asthma attacks"[Title/Abstract] OR "worsen*"[Title/Abstract] OR "hospitali*"[Title/Abstract] OR "emergency department"[Title/Abstract] OR "emergency departments"[Title/Abstract] OR "emergency visit"[Title/Abstract] OR "emergency visits"[Title/Abstract] OR "oral corticosteroid"[Title/Abstract] OR "oral corticosteroids"[Title/Abstract] OR "systemic corticosteroid"[Title/Abstract] OR "systemic corticosteroids"[Title/Abstract] OR "glucocorticoid*"[Title/Abstract] | 1,007,042 |
| **4** | "Disease Progression"[Mesh] | "Disease Progression"[MeSH Terms] | 236,025 |
| **3** | ("Asthma"[Mesh]) OR (asthma[Title/Abstract] OR bronchial asthma[Title/Abstract] OR asthmatic[Title/Abstract]) | "Asthma"[MeSH Terms] OR "Asthma"[Title/Abstract] OR "bronchial asthma"[Title/Abstract] OR "asthmatic"[Title/Abstract] | 224,207 |
| **2** | asthma[Title/Abstract] OR bronchial asthma[Title/Abstract] OR asthmatic[Title/Abstract] | "asthma"[Title/Abstract] OR "bronchial asthma"[Title/Abstract] OR "asthmatic"[Title/Abstract] | 202,368 |
| **1** | "Asthma"[Mesh] | "Asthma"[MeSH Terms] | 152,731 |
